# Socio-cultural components related to the cropping, harvesting, and consumption of guayusa (*Ilex guayusa* Loes) in Amazonian Kichwa communities

**DOI:** 10.1371/journal.pone.0349762

**Published:** 2026-06-02

**Authors:** Alejandra Espinosa Andrade, Wilson Vásquez-Castillo

**Affiliations:** 1 Universidad de Las Américas (UDLA), Quito, Ecuador; 2 Grupo de Investigación en Alimentos y Agroindustria (GIAA). Ingeniería Agroindustrial, Universidad de Las Américas (UDLA), Quito, Ecuador; Fort Valley State University, UNITED STATES OF AMERICA

## Abstract

Guayusa (*Ilex guayusa* Loes) is an Amazonian plant whose leaves contain chemical compounds with therapeutic and energizing properties. For the Kichwa nationality in Napo province, Ecuador, guayusa has traditionally been a fundamental part of their culture. Over the last decade, the export market for the plant has grown, leading to changes in its cropping, management, and use. Using qualitative methods, this study aims to identify and describe practices and beliefs regarding the planting, harvesting, brewing, and benefits of guayusa among Kichwa families today. The analysis shows that Kichwa families maintain some ancestral crop management practices and knowledge of guayusa’s benefits, although its consumption in family and ceremonial contexts has changed. Guayusa consumption now extends beyond familiar and ceremonial spaces and is prepared in various ways. Practices like dream analysis or punishment with tobacco and chili when drinking guayusa are now rare, while Guayusa’s benefits, including its energizing effects and ability to relieve sleepiness, laziness, body aches, and hunger, are highly valued. This study is a pioneer in considering the guayusa plant from a holistic perspective that takes into consideration not only the current socio-cultural aspects related to the plant but also its relationship with the chakra system.

## 1. Introduction

The study examines the management practices and contemporary beliefs surrounding the cultivation, harvesting, preparation, and perceived benefits of guayusa among Kichwa families in Napo, Ecuador. The research identifies: a) how Kichwa families currently manage guayusa within their households, including how it is cultivated, harvested, prepared, and consumed. b) the current cultural values and transformations associated with guayusa cultivation and use, and c) emerging research themes for a deeper understanding of Kichwa culture and its relation to guayusa.

This study documents contemporary plant management practices among Kichwa communities, contributing to a better understanding of the ongoing transformations of Indigenous knowledge systems. By examining how guayusa is cultivated, harvested, prepared, and understood within everyday life, the research provides valuable insight into the functioning of traditional agroforestry systems such as the *chakra*. In doing so, it not only records current practices and cultural meanings but also offers a broader understanding of how Indigenous knowledge adapts to changing social and environmental conditions. This contributes to global discussions on sustainable agroforestry, biocultural diversity, and the role of Indigenous practices in addressing current environmental challenges.

The Indigenous Kichwa nationality is present across all six Ecuadorian Amazon provinces, with the largest concentration in Napo, Pastaza, and Sucumbíos [3]. Today, the Kichwas are mainly settled in southern Napo. Their primary language is Kichwa, followed by Spanish. Traditional livelihoods include agriculture, hunting, fishing, fiber weaving, and ceramics [2,3].

Guayusa (*Ilex guayusa*), also called Waisa, Waysa or Wayusa in Kichwa language [4], is a shrub type plant [5] native to the Amazon region, which grows naturally as part of the ecosystem and, in some subtropical locations in cultivated form [6]. In South America it can be found in Ecuador, Colombia, Perú and Bolivia [1,7]. In the province of Napo-Ecuador, guayusa is found in the middle and lower zones of the province (300–1200 m.a.s.l), and is widely consumed by Kichwas, settlers and mestizos.

Archaeological evidence indicates the use of guayusa as early as 500 CE, with leaves found in a Tiahuanacoid tomb in highland Bolivia, suggesting its ceremonial and exchange value between lowland and highland regions [8]. Seventeenth-century accounts describe its mixture with narcotic plants such as Datura and Banisteriopsis, and tobacco in ritual and medicinal contexts [9,10].

In the Ecuadorian Amazon, guayusa cultivation is closely linked to the *chakra* production system, a family-managed agroforestry system that integrates ancestral knowledge through organic and biodiverse practices. As noted by Coral-Guerrero et al., the *chakra* is a living space for Indigenous families, providing food, income, and other essential resources [11]. This production model has demonstrated resilience despite market pressures and limited access to land [11]. It includes timber, fruit, medicinal, edible, and ornamental species, as well as endemic and domesticated fauna. The diversity of species within the *chakra* supports natural soil regeneration, prevents degradation, and ensures long-term food production [12]. Its spatial organization supports balanced production for household use and local trade while conserving agroecological and cultural traditions and preventing monoculture [2,13]. The chakra is primarily managed by the chakramama (chakra woman) with family participation [2,3,14]. Traditionally, mothers and grandmothers gift a guayusa plant to young women when they form new families and chakras, a custom passed down through generations [15]. Harvesting and brewing are mainly women’s tasks [1,2,4].

The cosmovision of the Amazonian Kichwa nationality of Napo and the ancestral practices derived from this cosmovision are extensive and have been described by several studies [3,4,15–23]. These explore diverse topics such as Kichwa mythology, rituals, language, the relationship between space and nature, clothing, economic activities, and their relationship with extractivism. Regarding guayusa, studies describe that before becoming a sacred plant, Guayusa was a divine being that instilled courage, lifted people’s spirits, stimulated wise decision-making, and conferred the joy of living and succeeding [15,24,25].

Several studies [3,4,15,26] describe the guayusa-taking ritual. The *guayusa upina* or *wayusaupina* ritual consists of getting up at dawn, making guayusa tea over an open fire, sitting around the fire, and drinking the infusion while the elders tell their stories, legends, and life experiences [23]. All members participate, and it is a space where reflection is sought, the day’s activities are planned, dreams are interpreted [4], and weavings are made using plant fibers to create utensils such as fishing nets, shigras (handmade woven bags), or ashanga baskets [7]. “The early morning *wayusaupina* functioned as a space for the transmission of knowledge; both women and men learned the practices they needed to develop for adult life and the forms of cooperation they were expected to carry out within their households” [27] p.125. In this ceremony, chili is also applied to the eyes of children and young people. This action is viewed as a punishment for behavior deemed inappropriate within the Kichwa family. It is also intended as a warning to the rest of the family to ensure that such behavior is not repeated [1,3].

The study by Radice et al. [7], describes the benefits attributed to guayusa by Amazonian Kichwa people, namely as a stimulant, stomach tonic, diuretic, flu remedy (in combination with other plants), cure for venereal diseases, blood cleansing, improved digestion, increased appetite and increased fertility in women, oral cleansing, and avoidance of insect bites. Some Kichwa even wash their dogs’ faces with guayusa so that they will dream and become good hunters [1]. It is also used for baths, washing the face, purifying the body, preventing aging [4,6,27] removing *chiki* (i.e., bad omen or misfortune) [28]. Guayusa leaves can also be mixed with ayahuasca [3], a ceremonial drink made from the stem and bark of the tropical liana Banisteriopsis caapi and other botanical plants. There are also mentions that guayusa leaves, mixed with orange, have been used as incense in ceremonies [29].

Recent studies show that the guayusa plant is rich in various chemical compounds that contribute to its medicinal properties and potential applications in pharmacology result [30]. Regarding the medical properties and primary chemical compounds in the guayusa plant, it includes phenolic compounds, such as chlorogenic acid and quercetin-3-O-hexose are the main representatives of hydroxycinnamic acids and flavonols, respectively, contributing to the plant’s antioxidant capacity, as evidenced by its ability to scavenge free radicals and reduce ferric ions [31–33]. Carotenoids, particularly lutein, are also present in significant concentrations in guayusa leaves [31,32]. Other important compounds identified in guayusa leaves include caffeine, theobromine, and 5-caffeoylquinic acid [34].The bioactive compounds in guayusa leaves, particularly in the ethyl acetate fraction, exhibit strong antioxidant and anti-inflammatory activities, indicating their potential therapeutic applications [32,33]. Given its biological properties and phytochemical compounds, guayusa is a plant of great interest to the food and pharmaceutical industries [31,35–39]; however, at present, guayusa is mainly used as an energy drink and herbal tea [25].

The national and international market for guayusa is rapidly expanding, and its economic importance is growing as a result [11,12]. The current supply of guayusa struggles to meet this demand, highlighting the need for sustainable cultivation practices [11,33,40]. Further studies are needed on the benefits of guayusa-based products and post-harvest management [41].

This present study addresses the lack of comprehensive research that integrates multiple dimensions of traditional plant use within Indigenous contexts. Existing studies often focus on isolated aspects, such as chemical composition, agronomic practices, or cultural significance, without providing a holistic analysis or a detailed account of how guayusa is managed at the local level. There remains a gap in understanding how these dimensions intersect, particularly in relation to current forms of management and ongoing sociocultural transformations. By examining guayusa through an integrated approach that considers its cultivation, use, and evolving cultural meanings, this study seeks to contribute to a more comprehensive understanding of traditional plant systems in contemporary contexts. Our findings, based on the perspectives of local producers, provide a detailed account of how guayusa is currently managed. They also indicate that guayusa is increasingly cultivated for national and international markets, while traditional practices such as dream interpretation and ritual punishment have become less common. The ways in which the infusion is consumed have evolved; nevertheless, its energizing and healing properties continue to be highly valued, even as commercialization challenges cultural continuity. Beyond contributing to the documentation of Indigenous practices and values, this study also opens new lines of inquiry into the cultural, social, medicinal, and economic dimensions of guayusa.

This study was made possible through the participation of local actors, who are the rightful holders of knowledge about guayusa and who placed their trust in us throughout the research process. Their willingness to share their experiences, practices, and perspectives was fundamental to the development of this work. We recognize that this knowledge originates from and belongs to them, and we aim to represent it with respect, accuracy, and responsibility.

## 2. Materials and methods

The methodology used was mainly qualitative. The techniques used were literature review, field observation, and semi-structured in-depth interviews with key actors belonging to the Kichwa nationality regarding the production and consumption of guayusa. Each technique is detailed below.

### 2.1. Bibliographic review

The bibliographic selection followed a systematic approach to ensure comprehensive coverage of scholarly work on guayusa (*Ilex guayusa* Loes). Searches in Scopus and Web of Science using the keyword “guayusa” identified literature on its ethnobotanical, biochemical, and agronomic properties. This was complemented by citation mining: backward (reviewing cited references) and forward (tracking newer citations) [42]. Scopus and Web of Science were chosen for their rigorous, interdisciplinary scope, while citation mining ensured inclusion of both foundational and recent studies, enabling a balanced and up-to-date synthesis of the literature.

### 2.2. Interviews

Eighteen semi-structured interviews were conducted with guayusa producers using the chakra system, eleven men and seven women, most affiliated with the Wiñak association. The Wiñak Association is an organization of Kichwa producers from the Ecuadorian Amazon, mainly in Napo, that promotes sustainable development based on the chakra agroforestry system. Its work combines agroforestry production, biodiversity conservation, and cultural strengthening, while supporting the commercialization of products such as cacao and guayusa under principles of sustainability and fair trade [43]. Sixteen of the eighteen interviewees are bilingual in Kichwa and Spanish. Kichwa is primarily used for everyday communication within the community and with neighboring villages, while Spanish is used to interact with mestizo populations. Most of the interviewees are between 30 and 45 years old and are actively involved in agricultural activities. All of them manage their own chakras, where guayusa stands out as one of the main crops. Those who work with the Wiñak association are also guayusa producers. (For more information about the interviewees see Appendix 1 – List of Interviewees).

Interviewees were selected for their direct involvement in guayusa cultivation, not for statistical representativeness, in line with qualitative research logic. Sampling followed the “saturation point” criterion, interviews continued until no new insights emerged. Topics included cropping, harvesting, consumption, family/ritual use, and transformations. Interviewees remain anonymous and are cited as E1, E2, etc., throughout the text. Of the interviewees, fourteen reside in the province of Napo and four in the province of Pastaza; however, the latter are affiliated with the Wiñak Association and work in Napo province. Three of the participants were interviewed in Quito during a university event.

### 2.3. Field observation and ethical guidelines

Five field trips were made during the period Sept 2022–Sept 2023. During these trips we visited five communities: Rukullacta, La Serena, Jatun Yaku, Tamia Urku and Flor del Bosque, located in Napo province. The objective of these visits was to conduct in situ interviews and observe the guayusa crops and the relationship between the producers and the plant.

All interviews were audio-recorded. Informed consent was obtained verbally to ensure that the process was culturally appropriate and non-intrusive. In this context, written consent procedures and formal documentation could have discouraged open participation and undermined trust between researchers and participants. Prior to recording each interview, the research objectives, procedures, and intended use of the data were explained orally to all participants in clear and accessible language, adapted to the local context. Participants were given the opportunity to ask questions and confirm their willingness to participate before the interview proceeded. In February 2024, a visit was made to present the results to the Kichwa producers in the city of Archidona in Napo province.

The authors adhered to the ethical principles established by the Ethics Committee for Research Involving Human Subjects of the Universidad de Las Américas (CEISH), as well as to the Declaration of Helsinki and the Code of Ethics of the International Society of Ethnobiology, which guided the research process. According to CEISH, the study is exempt from formal ethical review due to its observational design and the anonymized use of interview data, in accordance with current legal regulations (Certificate No. 2022-EXC-002). In addition, in 2023, institutional permission was granted by the General Coordinator of the Wiñak Association. This authorization confirms that the Association informed its members about the research and ensured that participants fully understood the process. It also specifies that participation was voluntary, that individuals could choose whether or not to take part in the interviews, and that they could withdraw at any time without providing justification.

## 3. Results

### 3.1. Planting and harvesting of guayusa

**Fig 1:**
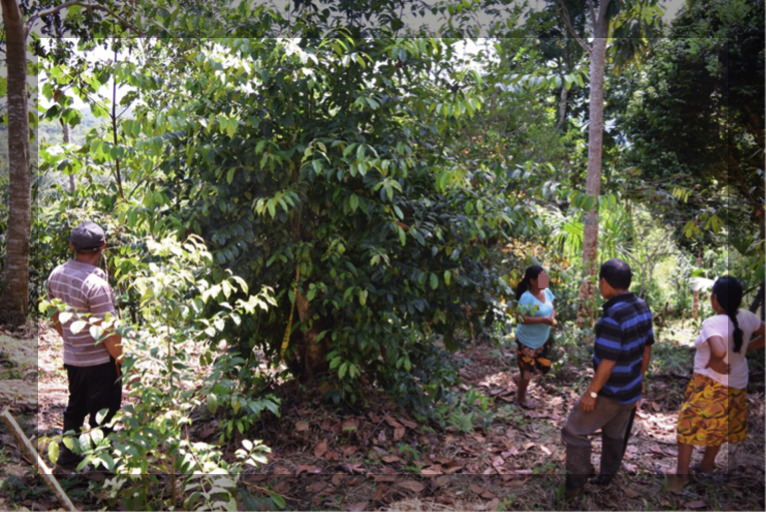
Kichwa community members in Napo Province, Ecuador, gathered around a guayusa tree (*Ilex guayusa*) in a chakra. Photo by Alejandra Espinosa (author). Reprinted under a CC BY license, with permission from Alejandra Espinosa, original copyright 2026.

Traditionally, Kichwa families have guayusa plants (Fig 1.) that they originally received from their grandparents or parents (E10, E14, E17). In other words, it is a plant with a hereditary component that is passed on from generation to generation. This passing on of the plant can be done in two ways. The first way is when parents give the plant to their children when they get married, and the new couple plant the guayusa in the new chakra where they will live. The second way is that fathers and mothers give a part of their chakra, which already contains a guayusa plant, as an inheritance to their sons and daughters (E16). For this reason, there is always a reference to the existence of at least one “mother plant” (E6, E14, E16), from which the families obtain shoots to plant next to the house and use for personal consumption (E6). Generally, it is not necessary to plant more guayusa plants because one or two plants at home are enough for their consumption (E6, E17). Previously “(...) guayusa was considered a sacred plant by the community, and they had one per community, sometimes two [...]. Everyone harvested the leaves and took them to brew it [...] because each community is a family” (E14). Since 2012–2013, they have started to reproduce and plant guayusa in the chakra with the aim of marketing it, with some families sowing it as a monocrop (E14). However, at present, due to a lack of buyers, some families have stopped harvesting the plants (E13).

A branch (35 cm long) of guayusa can be planted in the chakra at any time of the year and does not require any special care (E3, E6). Guayusa plants produce fruits that contain seeds; however, the most common method of propagating them is through selected cuttings from mother plants. Currently, in Napo, a chakra has a minimum extension of 0.25 hectares to a maximum of 1.5 hectares and can contain between 50–90 species of plants (E14), including yuca, plantains, peanuts, beans, chonta, and guayusa. Therefore, chakra is a business model incorporates the agroforestry characteristics of native products into their value, encompassing social, cultural, and environmental elements [13].

According to the producers, it is preferable to plant the guayusa in “black soil,” which is considered fertile, and not in “yellow soil,” which is considered poorly fertilized (E9). It is also preferable to plant it in a corner of the chakra, as when they grow, their roots become large and hard (E17). The soil must be cleaned (E3, E9, E10), which is done by hand, with a machete, or with a strimmer (E13). Some Kichwa producers mention that it should be planted without shade (E6) and others with “not much shade, but in some shade...” (E13). No specific distance from other plants is considered: “I plant this way in the spaces I have,” said one interviewee (E6). There are no considerations as to which species the guayusa should be planted next to, nor with which species it should be avoided. However, if guayusa is planted with trees around it, the leaves can get dirty: “It gets covered by epiphytes such as moss, it looks very ugly, the little black leaf can remain” (E10). The plant is not in danger of being invaded by animals or insects, and if it is sprayed with chemicals, it is damaged and not good for consumption (E13).

The Wiñak Association has established as one of its objectives to work in an environmentally responsible way, so it does not use chemicals or other synthetic inputs to prevent pests in its crops. For this reason, producers avoid planting guayusa near plants that use chemicals, as this could lead to contamination of the guayusa plant (E9). Diversification of species within the chakra system is believed to be beneficial for its growth, as the plant does not have to compete for nutrients. On the contrary, if guayusa is in monoculture, this may lead to leaf chlorosis and slower development (E14). The need to confirm this observation of the interviewees with scientific studies was mentioned (E14).

Regarding the care of the plant in the chakra, it does not require particular attention (E6). It is mainly handled by women (E14, E17), while the men perform the most physically challenging work, such as felling trees, carrying the plants, and clearing the land (E17). Generally speaking, according to cultural tradition, women are primarily responsible for looking after the Chakra. This is why they are known as Chakramamas, which means that they possess knowledge of the species found in the Chakra and how to care for them [44].

In terms of harvesting times, this can take place after four to five months (E5), seven months (E3), one to two years (E14), and even three years (E3). The time will depend on geographical location and soil fertility (E14). One plant can be harvested every 4 months (E3). Up to 3 sacks of leaves can be obtained from a single plant (E3); each sack is on average 25 kg. It has been stated that guayusa is harvested at any time of the day (E5, E10, E13), yet the morning is preferable, as this way it is possible to observe any damage to the leaves (E3). Only “clean” leaves are consumed or sold (E13).

It is mentioned that the day or time of harvest does not affect the benefits of the plant (E10). People harvest the “good leaves,” which should be clean, coarse-textured, and ripe (E12). They differentiate between the tender-young green leaf and the mature leaf, which is dark green and in the middle part of the branch (E13, E9). In terms of taste, some guayusa leaves are the same, and others have a more bitter taste (E10). The bitter taste depends on the infusion time and the number of leaves used. Shorter infusion time and fewer leaves result in a milder flavor. There is a difference between guayusa infusion made with younger leaves and guayusa infusion made with mature leaves (E12). Young guayusa leaves are those found at the tips of the branches, which are still growing and developing and have not yet reached maturity. These are light in color, softer and smaller. It was affirmed that it is better to drink an infusion of mature guayusa leaves (E9, E13), as the tender leaves are less flavorsome (E13). There is also a distinction between thin leaves and broad leaves (E6), the most “effective” being the thin leaves (E6).

The harvesting of guayusa can be carried out by any family member (E13), but it is mainly done by the women– the mother or grandmother (E10, E13, E18). Regarding the life span of the plant, it is considered a plant that “does not die,” that it is a perennial plant (E10, E14). E3 reports plants aged 56 years; however, older plants do exist [7]. In the study of genetic variability, plants of more than 100 years were found since the Kichwa communities indicate that they are three-generations old [14].

### 3.2. Brewing

**Fig 2:**
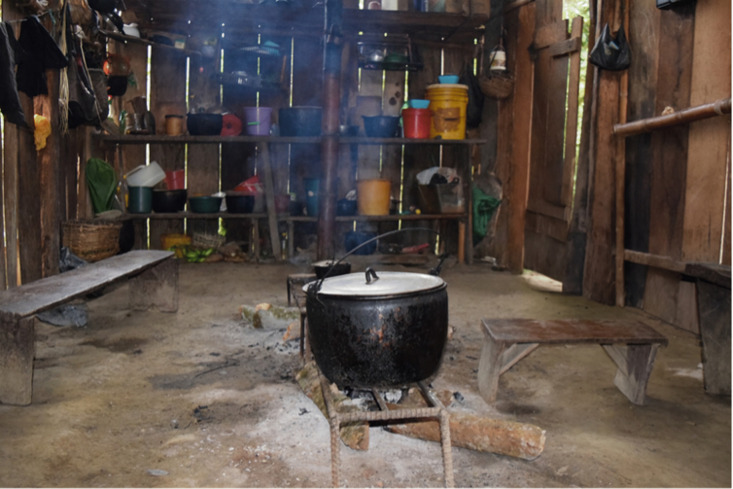
Firepit with tulpa in a traditional Kichwa house. Photo by the authors, 2022.

In the family sphere, a participant mentions that in the past “Grandmothers got up earlier. At two in the morning, three in the morning, they would light their little candle and the guayusa was always there (Fig 2). They had the guayusa [in] a little pot there. That was sacred to them; it was not to be touched. [...] So, they would get up at the appointed time, and, before going to sleep, they would light the tulpa and the guayusa there. The guayusa had to boil until she woke up. The guayusa would stay warm, all night long, yes. And that’s why the next day, when they drank the guayusa, it was black and bitter. So, they said that the guayusa was the good one, that is, as the companions said, it protected us from snakes, wasps, even from evil spirits [...]” (E15).

The above description summarizes one traditional way of brewing and consuming guayusa infusion. Other consumption methods, such as, for example, placing the leaves directly on the body or eating the leaf as food, are not mentioned at present. Although the making of the beverage may differ from family to family, the brewing methods follow distinguishable patterns, which are described below.

Once guayusa is harvested, the drink can be made using fresh or dried leaves, tender or ripe leaves (E17, E9). This depends on taste (E9, E1, E3). The ancestral and traditional way of drinking guayusa is mainly using dried leaves. There are different ways of drying the leaves. There are families that allow the leaves to dry for two or three days (E5), a week (E13), or a month (E10), either in the sun or inside the house, often the kitchen (E1, E10, E13). The most common way is to dry the guayusa leaves near the kitchen fire (E1, E6). Each family has a fire pit in their house of about 60 cm in diameter, near which a branch of guayusa is hung so that it gradually oxidizes with the heat of the fire (E1, E3, E6, E15). This “oxidized” guayusa is taken, boiled, covered, and drunk (E1).

To dry guayusa, some families make *sartas* (E16, E17): small holes are made in the freshly harvested tender guayusa leaves, through which a thread is passed and a sort of necklace of leaves is formed. The guayusa is then left to dry either indoors or outdoors (E13). When the leaves are dry, they can last between fifteen days and a month (E5).

Traditionally, the woman is in charge of making the guayusa drink (E1, E10, E18) and serves it to all members of the family, a ritual that is mainly passed from mother to daughter (E18). Nowadays, men also get involved in the brewing process (E1, E5, E10). To make guayusa infusion, a cup of leaves is placed in a pot of boiling water over the fire (E6, E7). The pot used exclusively to make the guayusa drink is called *waysamanga* or *tulpa* (E15, E16), and this is placed over the fire. The cup in which the guayusa is drunk is called a *pilche* (E1). The boiling time varies between 10 (E1), 20, and 30 minutes (E10, E13). When the smell of the leaves comes out, it means that the drink is ready (E10). If the leaves are dry, the smell comes out quickly (E6).

When the drink is made with green leaves, the leaves are harvested in the morning and boiled immediately, i.e., guayusa is made as a tea: the leaves are boiled in water and left covered for about ten minutes so that the compounds are extracted and the caffeine takes effect (E1, E10). This form of brewing is more recent: as mentioned above, the more traditional brewing method is by drying the leaves first.

Regarding the differences in the color of the drink, when it is made with mature and dried leaves, the water comes out a darker brownish-black color (E3, E9, 10) than when the leaves are green. Regarding the taste, with ripe and dried leaves, the taste is considered better (E5, E6, E13) and more bitter (E10). In terms of the effects produced by the different leaves, the interviewees affirm that these are the same (E3). However, in terms of intensity, it was mentioned that the more effective brew is “green” boiling (straight from the plant to the pot) due to the thermal shock and the longer boiling time (E1). On the other hand, it is also mentioned that “it is not the same to drink guayusa from an old tree than from a young tree; guayusa from the old tree is more loaded” (E1 in E9). Others mention that grandparents keep the guayusa leaves in the pot, which makes the drink stronger (E17).

There are families that make guayusa infusion the evening before and leave it to concentrate until the next day when they heat it and drink it (E1), and other families leave the guayusa in the pot all day (E6). When the guayusa is left to rest, it takes on a darker color and its effect is stronger (E6). This guayusa with a longer resting time is called guayusa tinta (E6, E18). A Kichwa informant mentioned that “at the moment of drinking the beverage, first the guayusa has to be well cooked, tinta [dark]. You rinse your mouth a little and spit it out. [...] Then, the infusion is usually drunk” (E18).

### 3.3. Consumption

There is no consensus on the age at which people start drinking guayusa. The age can vary between 1 year (E3), 2–3 years (E7), 6 years (E9), 8–10 years (E1), and 15 years (E5). It seems, then, that drinking guayusa has no known negative effects on minors; children drink it “when they want” (E7) and if they do not drink it, it is “because they do not want to” (E5). The custom of children drinking guayusa has been lost, mainly in the communities closest to the cantonal capital (E1). No restrictions are mentioned for drinking guayusa infusion; it can even be ingested during pregnancy (E1, E10). Animals do not drink the infusion or eat the guayusa plant (E3, E7).

Regarding the frequency and ways of drinking the beverage, the traditional/ancestral way is to drink it hot, without sugar (E1, E3, E17, E18), and in the early morning hours, when the day starts for Kichwa families. Their day usually starts between 1 and 5 a.m. (see Table 1). This first cup of guayusa can be drunk with bread (E7), and some families later drink a cup of chicha de yuca (E3, E4, E6, E13, E16, E17). After drinking guayusa, the day’s chores begin. In ancient times, and in some cases to this day, the men went fishing or hunting and the women stayed behind to do housework or traditional activities, such as making shigras (bags made from Amazonian plant fibres) (E11).

**Table 1:** Guayusa brewing and intake.

Subject	Interviewees responding to the topics
**Time of brewing and first drink**	
“Early morning”	E1, E6, E13
1–3 am	E4, E5, E6, E11, E15, E17
4–5 am	E6, E7, E9, E13, E16, E17
5–6 am	E7, E10, E17
Drunk at any time	E5, E7, E13
**Who makes it?**	
Women (mother/grandmother)	E3, E4, E10, E15, E16, E17, E18
Men	E9
Both women and men	E1, E5, E10
**Who drinks it?**	
Both men and women	E7
Women don’t like it, women don’t drink it, or women don’t drink as much as men.	E5, E6

Source: interviews with producers and members of Kichwa communities in Napo.

In terms of frequency of drinking, guayusa can be consumed at any time of the day (E5, E7, E13) and on occasions when you need to stay awake late at night (E3). During the day, it is drunk cold or sometimes heated (E6). Because of its energizing effects, some people prefer to drink it only once a day (E10, E17). It was mentioned throughout the day, people can take a pilche (a cup) as an energy booster (E1). It is the traditional morning drink of Kichwa families, who hardly ever consume coffee (E2, E3).

In the context of festivities, large pots of guayusa are made (E5), and it can be mixed with strong, homemade alcohol (aguardiente) and sugar (E3, E5, E17). Some families mix guayusa with lemon verbena, orange leaves, or cinnamon (E3), and when receiving guests in the house, this drink is also offered to them (E6). Children and younger people are the ones who prefer guayusa with sugar (E1, E3, E4, E7), but in general, this depends on each person’s preference (E3, E13).

### 3.4. Guayusa upina and ritual use

Guayusa upina is the time to drink guayusa. It is the time in the morning when, after or during the guayusa, the family interprets dreams and plans what will be done during the day (E1, E3, E4, E6). Dreams are considered to be signs to be taken into account for your future actions. For example, dogs symbolize snakes and danger, so if you dream about them, you have to be alert during the day (E1), but dogs are also positive signs that bring fortune.

Another ritual associated with drinking guayusa is giving advice and punishment to children (E1, E15, E16, E18). In the past, during guayusa upina, the eldest person in the family would gather all his children, and sometimes the grandfather (the rucuyaya) or an older relative or visitor, considered wise or a good hunter, would be invited (E1). It was common to put chili in the eyes of children who had misbehaved [45]. This ritual of putting chili in children’s eyes is called uchurina (E1). The person in charge of uchurina was the rucuyaya. The father or mother prepared different plants and had them ready for the grandmother or grandfather to perform uchurina (E1, E15, E16).

Another punishment that could be used in conjunction with chili was thrashing the child with nettles, and the children had to remain seated, listening to the advice (E1, E15). The idea was to have a moment of “training” for the younger ones by the adults. Another punishment associated with guayusa upina was putting tobacco up the nose (E13). Tobacco, which is also sown in the chakra, was harvested and left to dry by the fire for 10–12 days (E15). Then, the tobacco was cut, crushed, and soaked in water overnight. Some people boiled it. The remaining water is put up the nose, which causes coughing and crying (E1, E15). Tobacco is still used in this way today, as it is thought to be beneficial for treating flu (E15).

Currently, the guayusa upina ritual is also performed at the community level (E6, E16, E18). In some communities, they also impose the punishment of chili and tobacco on community leaders who are doing their work badly (E6, E16), or they also take their children to this community ceremony. There are also punishments with nettles or a guayusa whip (E16). After the ritual, there are drum sounds (E6), and there may also be a pambamesa where fruits and typical local dishes are served. Sometimes, there are also bundles of herbs, such as lemon verbena, for people to take with them (E18). The ritual of guayusa upina has transcended Kichwa communities and has become a kind of festival to attract visitors to the area. If there is a community celebrating its anniversary, the guayusa upina ceremony is performed in a dramatized style (E15). It has also taken on a political dimension; authorities hold events where the guayusa upina is performed as a big show/festival with music (E1).

The use of the guayusa plant is also associated with shamanic ceremonies. Shamans take yagé (or ayahuasca) and then rinse their mouths with guayusa (E1). Others say that guayusa is drunk before yagé (E6) or guayusa is mixed with yagé (E2, E6). Yagé or Ayahuasca is a sacred plant that is used only by shamans (E10) and allows them to reach other states of knowledge. Other plants with which yagé is mixed are amirunca or amelronca (E2).

### 3.5. Perceived present-day effects and benefits of the plant

Guayusa is perceived as the pillar of Kichwa culture, a medicinal and sacred plant that has been maintained for generations (E15, E16). “Without guayusa, we are not Kichwa [...]; without guayusa, we cannot live,” commented one interviewee (E16). In ancestral times, this plant was only used by the yachak or shamans, those who had the power, the energy of the forest, and the water (E14). One of the people interviewed said:

...I think guayusa controlled the mind, that is, the brain. It was, for example, I remember that my father, when we were already sick, we never went to the health center or hospital, nothing, nothing. Before, it was only the shaman, shaman, shaman. That’s who our doctors were, the shamans. So always when [the shaman] came, he would say, ‘You have to drink guayusa; give him strong guayusa.’ He would drink it, blow on the head, the headache would go away...(E17).

One informant mentioned that the chiriguayusa (Brunfelsia grandiflora), described as a bush with smaller leaves [46], has stronger effects and can only be taken when one has knowledge of ancestral medicine or if one has some power, like that of the shamans (E18).

The chiriguayusa could also be mixed with other plants to be used as a diuretic infusion and to cure blood-related diseases (E18). The main properties attributed to the intake of guayusa infusion are described in the following Table 2.

**Table 2:** Properties of the guayusa infusion according to Kichwa interviewees.

Properties	Mentioned by interviewees
Eliminates sleepiness/laziness	E2, E3, E4, E5, E6, E7, E10, E11, E13, E17, E18
Gives energy/strength	E5, E6, E14, E4, E7, E15, E18
Removes body aches/keeps you healthy	E2, E4, E5, E6, E7, E10, E13
Scares away snakes/makes snakes afraid	E1, E2, E3, E10, E13, E15, E16, E17, E18
Scares away wasps/no wasp stings	E1, E2, E3, E15, E16
Takes away hunger	E4, E5, E6
Makes you happy	E15
Helps you to be aware of animals	E17
Helps in planning activities/work	E3, E5
Increases concentration	E5
Increased virility/desire to have sex	E1
Increased female fertility	E1, E14
Repels evil spirits	E1, E15
The body needs guayusa	E3
Helps eyesight	E15
Beneficial for pregnant women	E15
Helps the stomach/digestion	E1, E15
Repels “tigers”	E15, E17
Keeps your teeth clean	E15
Regulates menstruation	E16
Diuretic	E18
Helps cure blood-related diseases	E18

Source: interviews with producers and members of Kichwa communities in Napo.

As can be seen, the most mentioned properties are currently related to the provision of energy and the elimination of sleep, laziness, body aches, and hunger. Because of these benefits, guayusa is drunk at the beginning of the day, before going out to work (E3) and for jobs that require energy, which in Kichwa communities have traditionally been carried out by men, such as fishing and hunting, and which also require walking long distances (E1).

Unlike coffee, guayusa does not produce addiction or effects of alteration or agitation in the body, even if taken in large quantities (E1, E9). Benefits related to scaring off animals such as snakes, wasps, and “tigers” are also mentioned. To a lesser extent, other properties are mentioned, including making you cheerful, helping you plan activities, aiding concentration, increasing virility in men and fertility in women, warding off evil spirits, improving eyesight, improving digestion, keeping teeth clean, regulating menstruation, curing blood-related diseases, and as a diuretic.

Because of these multiple properties, guayusa is still considered a medicinal plant today. During the COVID-19 pandemic, in order to stay healthy, Kichwa communities did not stop drinking guayusa. It was constantly ingested “so that the throat would not dry up” (E6), along with other plants such as ginger, lemon, chuchuhuaso, huranga, matico (spiked pepper), sangre de drago, llustunda muyu, and uña de gato (cat’s claw) (E6, E14). It was mentioned that the uptake of guayusa also depends on the communities, some of them drink the guayusa for hunting, others for fishing, depending on which one is the main activity of the community. In the case of the Achuar people, it is mentioned that they take guayusa in large quantities to induce vomiting and cure their stomachs (E1). This coincides with studies mentioning that in the past, people in Ecuador have been described as using feathers to induce vomiting after having ingested guayusa [1]. The study of Radice et al. [7], also points out daily purging as part of the traditional, magical and ritual uses of guayusa, and the study of Schultes from 1972, mentions that, at the time of the study, indigenous people wake up in the morning and drink guayusa to make them vomit [8].

## 4. Discussion

The chakra is a farming system practiced by the Kichwa indigenous people of northern Ecuador’s Amazon region, which ensures their food security thanks to the wide variety of crops they grow [47,48], the size depends of communal land, its location and the number of the families in the community [47,49]. It is a key agroforestry model for Kichwa culture and environmental sustainability in the Ecuadorian Amazon, but it is increasingly under pressure. Climate change, deforestation, market demands, population growth, and land scarcity have transformed its dynamics. Sociocultural shifts (such as youth migration, aging knowledge holders, and reduced land availability) further challenge its continuity [48]. In terms of planting and harvesting guayusa, the analysis provided in this study is valuable as it offers a detailed description of how the Kichwa people receive the guayusa plant, place it in the chakra, and determine the parameters for its cultivation and harvest. This contributes to the documentation of local knowledge, which may be valuable for further research on traditional cropping systems or the history of the Kichwa people.

In general, guayusa harvesting in its traditional and ancestral form has been gradually modified. In the past, there were very few guayusa plants on the chakra, since they were grown solely for family consumption. Most guayusa plants grown in the chakras are propagated vegetatively (from cuttings) from selected parent plants. This reduces the genetic diversity of the species and makes it more vulnerable to biotic and abiotic factors [1,29]. The first significant modification is related to the planting of guayusa for commercial purposes, preserving the chakra production system. Apart from drinking guayusa at the family and community level, families now also perceive it as a source of income (E14). This is validated by UDLA and GIZ [14] and Prefectura del Napo [2]. This implies a modification in the chakra production system, as guayusa occupies a larger physical space for cultivation. Furthermore, it entails a reconfiguration in the local and family economy: on the one hand, the commercialization of guayusa provides additional income for the families; on the other hand, if commercialization is unsuccessful, the plant remains in the chakra without being harvested [25]. The expectation of commercializing guayusa leads Kichwa families to allocate part of their land to monoculture, intercropping, a living fence, among other forms [43]. The producers interviewed perceive this as a risk that could change Indigenous customs and cosmovision (E14), while plowing the land, caring for the chakra, and learning the Kichwa language are perceived as essential to Kichwa culture [43] which coincides with what is indicated by E16.

Agricultural activities geared toward the market encourage monoculture, eliminating or reducing plant diversity and, consequently, wildlife, thereby impacting the environment and, at the same time, transforming society’s relationship with its surroundings [50]. Guayusa monoculture at high densities is not viable, as it requires interplanting with other species in order to grow, due to the symbiotic relationship that develops between them, and also because of the organic certification required for its sale [27,51]. As a way to counteract this widespread expansion of monocultures, initiatives led by local governments and international organizations aim to sustain and strengthen the chakra system. One example is the Amazonian Kichwa Chakra Seal, promoted by the Chakra Corporation, which seeks to recognize and differentiate products grown under this model in national and international markets. By incorporating ancestral knowledge and practices, the seal adds value to these products and enhances their competitiveness, while supporting the long-term livelihoods of Kichwa families [44,48]. The Chakara Seal represents a management model that emphasizes production, environmental, and cultural aspects aimed at fair trade and seeks to have these recognized in commercial relationships [27,52].

In terms of management, women are primarily responsible for the chakra and guayusa harvesting; however, these roles have become more flexible in recent years [43]. In terms of the way the guayusa tea is made, guayusa used to be boiled in water using chonta or palm wood as fuel for the fire. Gradually, families have started using industrial cookers, which, according to Kichwa perceptions, have changed the taste of the drink (E6). Nowadays the infusion is made using not only dried leaves, but also fresh, tender, or ripe leaves (E17, E9). Some Kichwa families (E16) also make guayusa infusion using small bags processed by the Wiñak Association [43].

In the past, for healing purposes, guayusa was drunk very strong and bitter. Nowadays guayusa infusion is drunk hot or cold (E6) and at any time (E1); some people prefer to drink it with sugar (E10) and others with sugar or panela (raw cane sugar) and lemon, this was also reported by De la Torre [24]. All these changes are part of the “domestication of guayusa” (E1). Throughout Napo province, it is common for restaurants to offer guayusa as the main drink, which is drunk like any other (E17). Nowadays, almost all beverages on the market have high levels of sugar, so the population is also used to sweetened guayusa. In the case of children, they prefer juice to traditional drinks, such as guayusa or chicha (E13). Young Kichwa people who live outside the community for work or studies stop making guayusa in their new home. However, when they return to their community, they drink guayusa and sometimes take leaves with them to brew in their new home.

In terms of the ritual of drinking guayusa, the time for drinking it is more flexible. It is no longer limited to the first hour of the day but can be done at any time. This flexibility is mainly found in the urban area, where guayusa is served in shops and restaurants, together with food. Regarding the tradition of sharing and analyzing dreams, this practice used to be carried out mainly by grandparents in the past [24]. This is because guayusa allows to connect mind, body, and spirit, bringing various benefits such as more energy, concentration, and vigilance [15} (Franco et al. 2018). Although some families still maintain it, the custom is on the decline.

At the community level, guayusa upina has also taken on a more commercial aspect; there are now events organized by Kichwa people using the theme of guayusa to attract visitors, such as the “gran Guayusazo Bailable” or the “Guayusa Warmi” contest, where at the end of the event, the winner distributes guayusa among those present, which is also reported by Andy et al. [1].

These transformations are driven by multiple, interconnected factors. First, shifts in economic structures at household, national, and global levels have altered production priorities, encouraging a greater orientation toward market participation. In this context, guayusa has increasingly emerged as a strategic crop, offering new income opportunities for Kichwa families through its integration into national and international value chains. Second, processes of urbanization and globalization have reshaped consumption patterns and the relationship between communities and their surrounding environment, contributing to changes in traditional practices and knowledge systems.

At the same time, structural constraints persist. Many Kichwa communities face limited access to land, as communal territories are insufficient to support new generations, leading to fragmentation and reduced availability of space for traditional agroforestry systems such as the chakra. Economic challenges are also significant, as products cultivated within the chakra are often sold at low prices in urban markets [2], limiting their contribution to household livelihoods.

In this context, the commercialization of guayusa presents both opportunities and tensions. On the one hand, it can help alleviate some of these challenges by providing a more stable source of income and enhancing the economic value of agroforestry systems. On the other hand, increased market integration may contribute to shifts in production practices and cultural meanings, potentially affecting the transmission of traditional knowledge and the social organization surrounding guayusa cultivation and use.

The people interviewed highlighted the importance of maintaining the tradition of using guayusa, as they consider it part of their essence and Kichwa culture. Specifically, the Wiñak Association seeks to promote the Kichwa culture so that it is not “lost” and guayusa is part of this promotion, we share this criteria indicated by Noriega et al., [53]. In 2023, the National Assembly declared March 25th as National Day of Guayusa Upina [54] as a way of maintaining the customs of the Indigenous peoples and nationalities of Ecuador.

Regarding the limitations of the present study, the main one may be that most of the interviewees are part of the Wiñak Association. This association has its own particular point of view regarding the management of the chakra system and guayusa production. The association promotes responsible and sustainable production, as well as a discourse related to the importance of maintaining the Kichwa culture and cosmovision. For these reasons, this study should be taken as a specific vision of a group of Kichwa people, which is not generalizable to the entire Amazonian Kichwa nationality.

## 5. Conclusions

The analysis describes the management of guayusa plant among the Kichwa people and, it also shows how Kichwa families currently maintain some ancestral practices related to the management of the plant and, at the same time, these practices have undergone transformations in relation to the form of family consumption and ceremonies. From the interviewees, they face a challenge in maintaining the traditional use of guayusa among the younger generations, as for them, it is important to preserve Kichwa identity. Practices such as the analysis of dreams or punishment with tobacco and chili when drinking guayusa are not frequent. Similarly, guayusa is being planted not only for family consumption but also to be sold in national and international markets. In terms of the plant’s benefits, guayusa is still considered a medicinal plant today, they mainly emphasize the endowment of energy and the elimination of sleepiness, laziness, body aches, and hunger, which are highly valued.

The analysis presented opens new questions about guayusa and possible future research topics, such as 1) risks of the expansion of guayusa cultivation in the chakra system (as a culturally and environmentally sustainable space); 2) relationship between guayusa and species diversification (chakra system); 3) relationship between the age of the guayusa plant and its effects; 4) relationship between the maturity stage of the leaf and its effects; 5) other forms of consumption (pills, syrups, ointments, etc.); and 6) the economic and environmental impact of guayusa monoculture on Kichwa families.
